# Supplementary material for: Applied Clinical Tandem Mass Spectrometry-Based Quantification Methods for Lipid-Derived Biomarkers, Steroids and Cannabinoids: Fit-for-Purpose Validation Methods
Source: Biomolecules. 2023 Feb 17;13(2):383. doi: 10.3390/biom13020383 (PMC9953102; doi:10.3390/biom13020383)

Table S1. Source (supplier) and purity of reference standards (RS) and internal standards (IS) for steroids (STs) (A) and cannabinoids (CBs) (B).

**A. STs**	**Source**	**RS purity**	**IS purity**
PREG	Cerilliant	99.84	-
PREG-d4	CDN - Isotopes	-	98
PROG	Cerilliant	99.35	-
PROG-d9	CDN - Isotopes	-	98.7
17OH-PROG	Cerilliant	98.25	-
17OH-PROG-d8	Cerilliant	-	99.9
ALLO	Steraloids	95	-
ALLO-d4	CDN - Isotopes	-	99.1
EPIALLO	Steraloids	NP	-
EPIALLO-d4	CDN - Isotopes	-	98.7
EPIAPRAG	Steraloids	NP	-
PRAG	Steraloids	NP	-
PRAG-d4	CDN - Isotopes	-	98
3α,5α-THDOC	Steraloids	98	-
3α,5α-THDOC-d4	CIL	-	95
DHEA	Cerilliant	99.87	-
DHEA-d5	Cerilliant	-	99.65
TESTO	Cerilliant	99.54	-
TESTO-d3	Cerilliant	-	99.9
DHT	Cerilliant	99.80	-
DHT-d3	Cerilliant	-	95.63
**B. CBs**	**Source**		**IS purity**
2-AG	Cayman	100	-
2-AG-d5	Cayman	-	≥ 99
AEA	Cayman	100	-
AEA-d4	Cayman	-	≥ 99
OEA	Cayman	98	-
OEA-d4	Cayman	-	≥ 99
PEA	Cayman	100	-
PEA-d4	Cayman	-	≥ 99
THC	Sigma	98.51	-
THC-d3	Sigma	-	99.5
11COOH-THC	Sigma	99.71	-
11COOH-THC-d3	Sigma	-	99.5
11OH-THC	Sigma	95.55	-
11OH-THC-d3	Sigma	-	98.9

NP: not provided by the supplier

Table S2. Concentrations of stock and working solutions (WS) of the reference standards for steroids (STs) (A) and cannabinoids (CBs) (B).

A. STs

Reference standards	Stock solutions	WS-1 (ng/µl)	WS-2 (pg/µl)	WS-3 (pg/µl)
PREG	10 µg/µl	10	80	5
PROG	10 µg/µl	100	1000	250
17OH-PROG	1 µg/µl	2	80	1
ALLO	1000 ng/µl	2	16	1
EPIALLO	1000 ng/µl	2	16	1
EPIPRAG	10 µg/µl	2	16	1
PRAG	10 µg/µl	0,5	4	0
3α,5α-THDOC	2 µg/µl	20	160	10
DHEA	1000 ng/µl	10	80	5
TESTO	10 µg/µl	10	60	10
DHT	1000 ng/µl	10	80	5

B. CBs

Reference standards	unit	Stock solutions	WS-1	WS-2	WS-3	WS-4
2-AG	pmol/µl	1000	50	10	0.5	
AEA	pmol/µl	1000	50	10	0.5	0,1
OEA	pmol/µl	500	100	10	0.5	
PEA	pmol/µl	500	100	10	0.5	
THC	ng/µl	1000	10	0,5	0.05	
11COOH-THC	ng/µl	10	0,5	0,05		
11OH-THC	ng/µl	10	0,5	0,05		

Table S3. Concentrations of stock and working solutions (WS) of the internal standards for steroids (STs) (A) and cannabinoids (CBs) (B).

A. STs

Internal Standards	Stock solutions	WS (ng/µl)
PREG-d4	1000 ng/µl	10
PROG-d9	10 µg/µl	100
17OH-PROG-d8	2 µg/ml	2
ALLO-d4	1000 ng/µl	2
EPIALLO-d4	2000 ng/µl	2
PRAG-d4	10 µg/µl	1
3α,5α-THDOC-d3	10 mg/ml	20
DHEA-d5	100 µg/ml	10
TESTO-d3	100 µg/ml	10
DHT-d3	100 µg/ml	10

B. CBs

Internal Standards	Stock solutions	WS
2-AG-d5	2.6 nmol/µl	10 pmol/µl
AEA-d4	2.8 nmol/µl	1 pmol/µl
OEA-d4	3 nmol/µl	10 pmol/µl
PEA-d4	3.3 nmol/µl	10 pmol/µl
THC-d3	100 µg/ml	0,2 ng/µl
11COOH-THC-d3	100 µg/ml	1 ng/µl
11OH-THC-d3	100 µg/ml	0,2 ng/µl

Figure S1. Chromatographic separation for target STs and respective deuterated analogues (IS) of mid QC samples measured by GC-MS/MS in negative SRM mode. Retention time (RT) and area under the curve (MA) are given for each peak integration (dark gray).

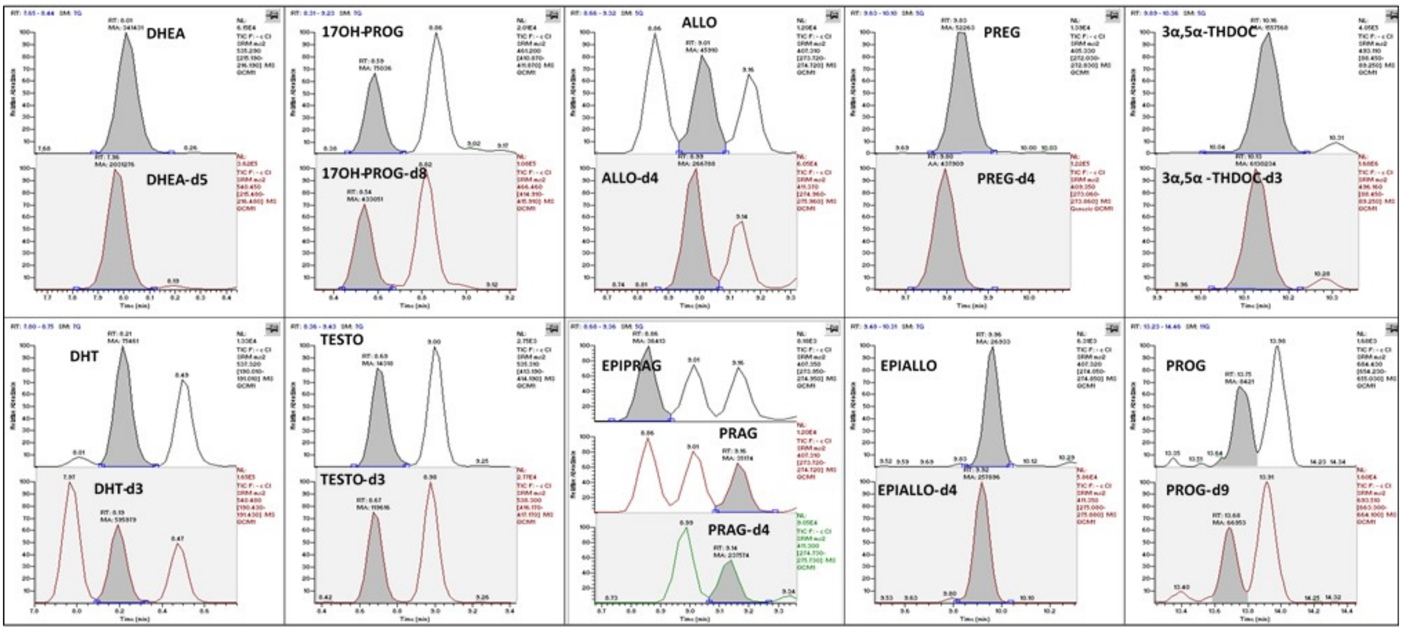

Figure S2. Chromatographic separation for target CBs and respective deuterated analogues (IS) of mid QC samples measured by LC-MS/MS in positive SRM mode. Retention time (RT) and area under the curve (MA) are given for each peak integration (dark gray).

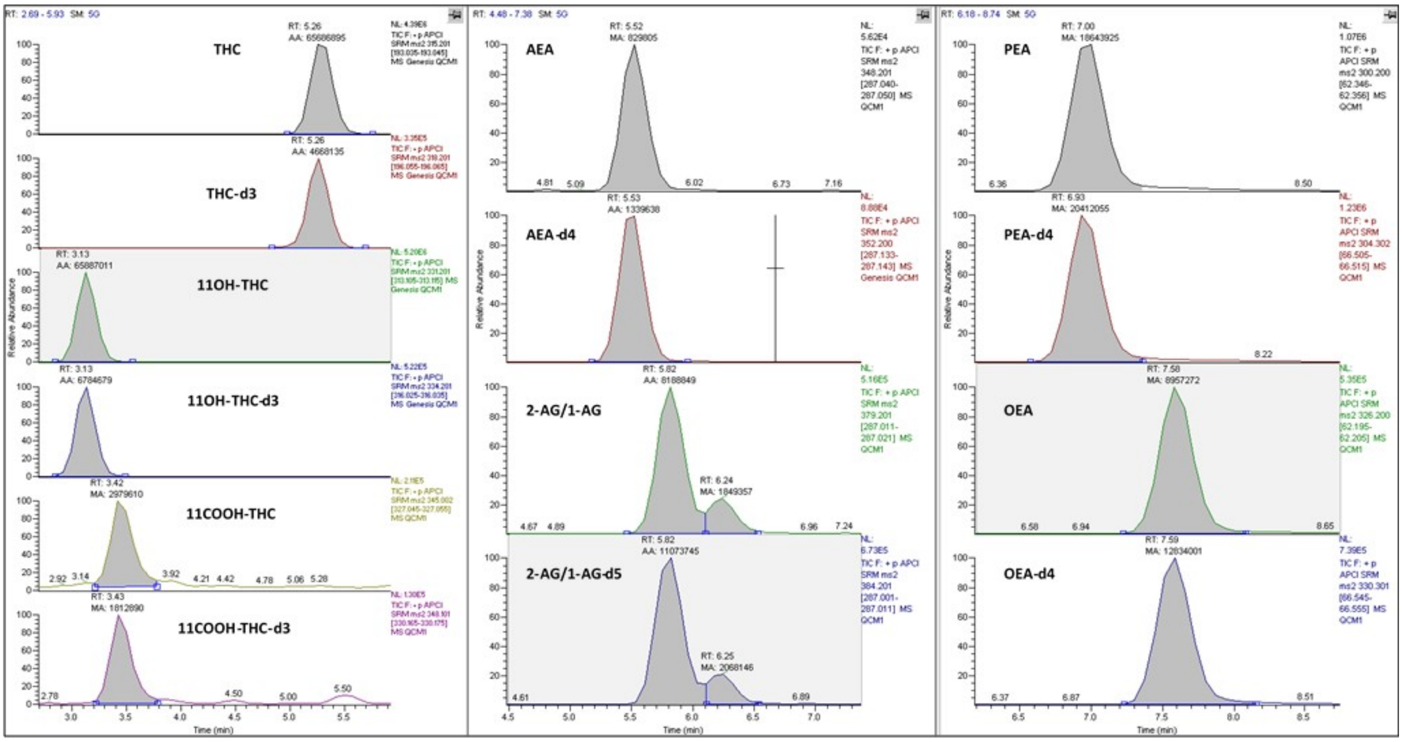

Supplement: Supplementary file 1 [file biomolecules-13-00383-s001.zip › biomolecules-2149605-supplementary.pdf]
